# Supplementary material for: Room-Temperature Synthesis of Carbon Nanochains via the Wurtz Reaction
Source: Nanomaterials (Basel). 2025 Mar 6;15(5):407. doi: 10.3390/nano15050407 (PMC11901694; doi:10.3390/nano15050407)
Supplement: Supplementary file 1 [file nanomaterials-15-00407-s001.zip › nanomaterials-3500301-supplementary.pdf]

Supplementary Information

Room-Temperature Synthesis of Carbon Nanochains via the Wurtz Reaction

Juxiang Pu, Yongqing Gong, Menghao Yang * and Mali Zhao *

Interdisciplinary Materials Research Center, School of Materials Science and Engineering, Tongji University, Shanghai 201804, China; 2230661@tongji.edu.cn (J.P.); 2331571@tongji.edu.cn (Y.G.)
* Correspondence: menghaoyoung@tongji.edu.cn (M.Y.); mali_zhao@tongji.edu.cn (M.Z.)

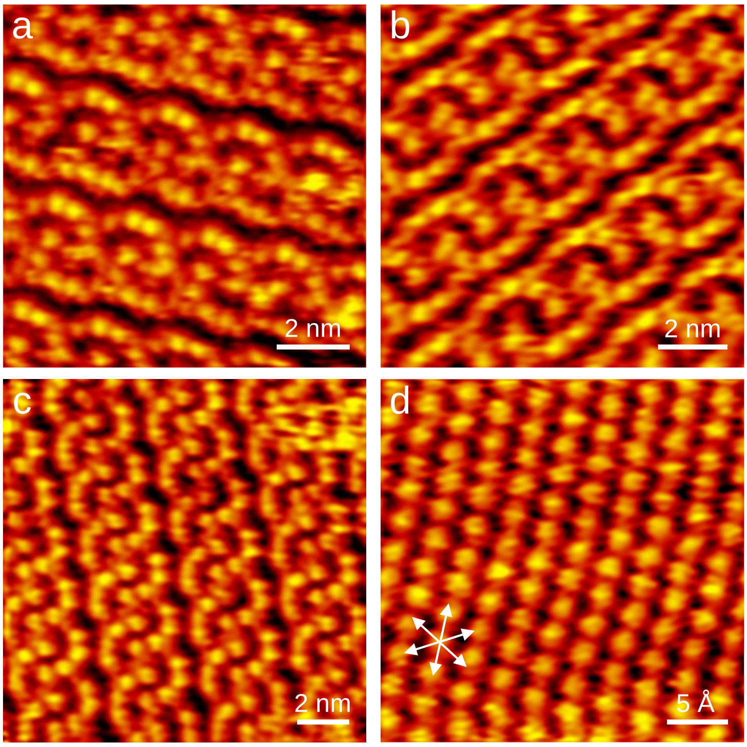

Figure S1. (**a**), (**b**) and (**c**) STM images of self-assembled BBMBN molecules along three different directions after deposition on Ag(111) at 135 K. (**d**) Atomic-scale STM image of Ag(111) substrate.

Isomer (chemistry)	R-isomer	S-isomer
Structural model	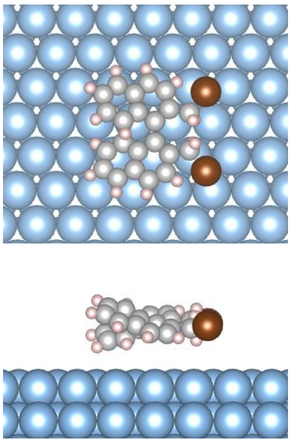	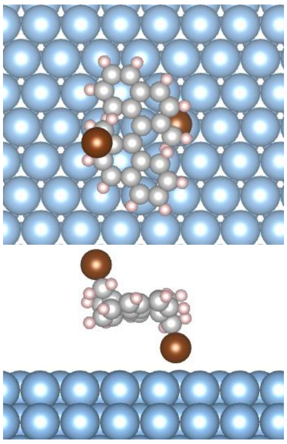
Total energy	0.22 eV	**0 eV (Preferred)**
Simulated STM image	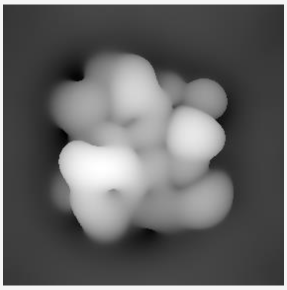	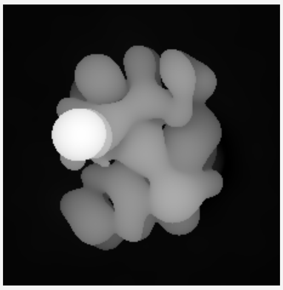

Figure S2. Comparison of cis-BBMBN (R-isomer) and trans-BBMBN (S-isomer) molecules on Ag(111).

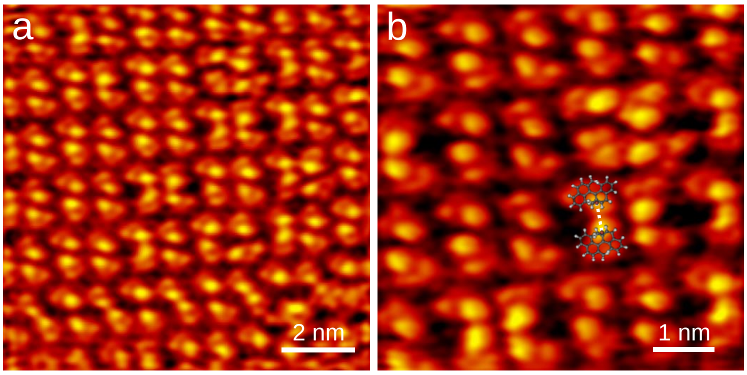

Figure S3. (**a**) Large-scale and (**b**) zoomed STM images of BBMBN* molecules after 135 K deposition followed by annealing at 370 K for 10 min. Scanning condition: V_t= −1.25 V， I=0.6–0.9 nA. A model of BBMBN* molecules and halogen bond (white dashed line) between adjacent molecules are overlaid on the corresponding STM image, and detached Br atoms are indicated by white dashed circles.

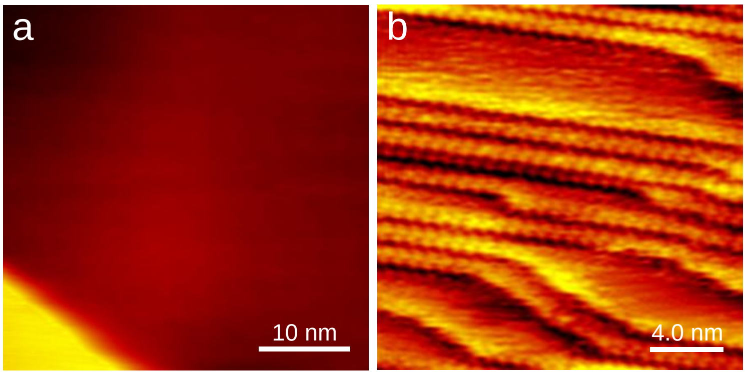

Figure S4. (**a**) STM image of clean Ag(111) surface before molecular deposition. (**b**) STM image of carbon nanochains along step edges after depositing BBMBN molecules at RT (300 K) with subsequent annealing sample at 300 K for 12 h.

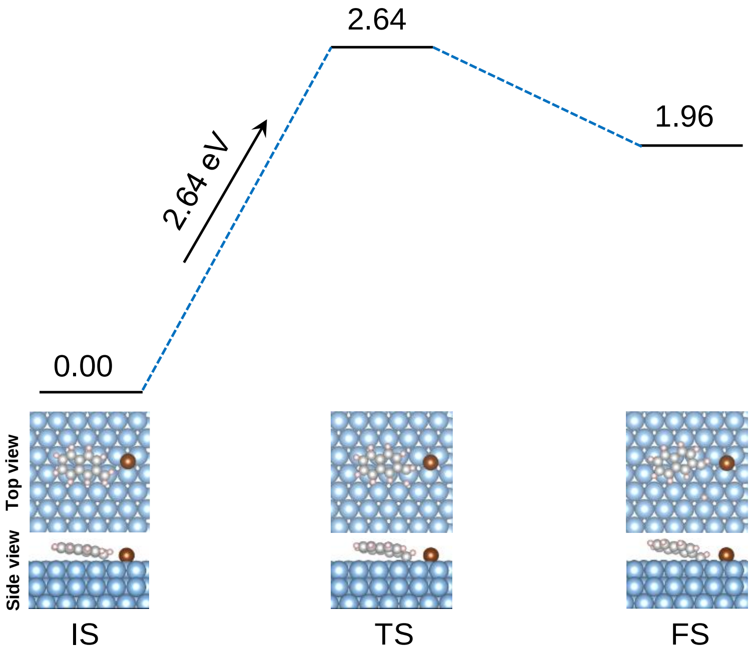

Figure S5. The DFT-calculated energy diagram for the C–H bond breaking of 2–(Bromomethyl)naphthalene on Ag(111) is shown, below are the structural configurations of the initial states (ISs), transition states (TSs), and final states (FSs).

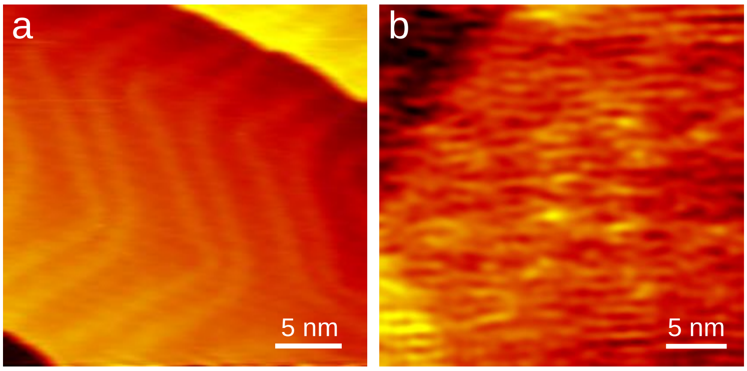

Figure S6. STM images of Au(111) surface before (**a**) and after (**b**) BBMBN molecular deposition at room temperature.
